# Clinician awareness of tetanus-diphtheria vaccination in trauma patients: a questionnaire study

**DOI:** 10.1186/1757-7241-20-35

**Published:** 2012-05-15

**Authors:** Young-Hoon Yoon, Sung-Woo Moon, Sung-Hyuk Choi, Young-Duck Cho, Jung-Youn Kim, Young Ho Kwak

**Affiliations:** 1 Department of Emergency Medicine, Korea University College of Medicine, Seoul, South Korea; 2 Department of Emergency Medicine, Seoul National University College of Medicine, Seoul, South Korea

**Keywords:** Tetanus, Tetanus-diphtheria, Prophylaxis

## Abstract

**Background:**

Most trauma patients visit the hospital via the emergency department. They are at high risk for tetanus infection because many trauma patients are wounded. Tetanus immunity in the Korean population has been revealed to be decreased in age groups over 20 years old. It is important for emergency physicians to vaccinate patients with the tetanus booster in wound management.

**Methods:**

Questionnaires were sent to the directors of the emergency departments of resident training hospitals certified by the Korean Society of Emergency Medicine.

**Results:**

Two thirds of the emergency department directors surveyed reported applying tetanus prophylaxis guidelines to more than 80% of wounded patients. However, about 45% of clinicians in the emergency departments considered giving less than half of the wounded patient tetanus booster vaccinations, and there were no distinct differences in tetanus booster vaccination rates among different age groups. Most emergency physicians are familiar with tetanus prophylaxis guidelines for wound management. However, more than half of the emergency department directors reported that the major reason for not considering tetanus-diphtheria vaccination was due to assumptions that patients already had tetanus immunity.

**Conclusion:**

Attitude changes should be encouraged among emergency physicians regarding tetanus prophylaxis. As emergency physicians are frequently confronted with patients that are at a high risk for tetanus infection in emergency situations, they need to be more informed regarding tetanus immunity epidemiology and encouraged to administer tetanus booster vaccines.

## Background

The incidence of tetanus has been decreasing due to widespread use of the tetanus vaccine. Although the incidence of tetanus is low in developed countries, the worldwide incidence of tetanus is 1 million cases per year and the mortality rate is between 20% and 45% [1].

In South Korea, childhood immunization for tetanus began in 1954; the tetanus vaccination coverage rate in children was 39.3% in 1968 and 86.7% in 1977 [2]. The diphtheria and tetanus toxoids and acellular pertussis vaccine (DTaP) was introduced in 1982 and the DTaP coverage rate in children has been over 90% since 1988 [3].

The Korea Centers for Disease Control and Prevention and the Korean Society of Infectious Disease recommend that children get 5 doses of DTaP, one dose at each of the following ages: 2, 4, 6, and 15–18 months, and 4–6 years. They should also get a tetanus toxoid with lower doses of diphtheria and acellular pertussis than DTaP (Tdap) or tetanus-diphtheria vaccination at 11–12 years and tetanus-diphtheria vaccination every 10 years from the age of 19 years. One of every three tetanus vaccinations between 19 and 39 years should be Tdap instead of tetanus-diphtheria vaccine [4,5].

Because immunity to tetanus decreases over time, the Centers for Diseases Control and Prevention (CDC) recommends that adults receive booster vaccines every 10 years [6]. However, the tetanus booster vaccine was not launched in South Korea until 2004, and is still not widely used due to lack of awareness. Korean emergency physicians (EPs) are expected to follow the CDC recommendation of tetanus prophylaxis in wound management; the CDC recommends that even adult patients with clean minor wounds should receive tetanus-diphtheria vaccinations if their last booster was over 10 years prior [7].

Currently, the tetanus immunity in the Korean population has been revealed to be decreased in age groups over 20 years old and only 10% of Koreans over 40 years old have tetanus antibody levels over 0.1 IU/mL [8]. Tetanus cases nearly disappeared in South Korea in the 1980s and 1990s. However, there have been about 10 tetanus cases per year since 2000 [9].

Most trauma patients visit the hospital via the emergency department (ED). They are at high risk for tetanus infection because many trauma patients are wounded. It is important for clinicians to vaccinate patients with the tetanus booster after obtaining tetanus vaccination histories. We investigated EPs’ awareness of the need for tetanus-diphtheria vaccination of trauma patients.

## Methods

Questionnaires were developed and sent to the directors of the EDs of the resident training hospital certified by the Korean Society of Emergency Medicine. The questionnaires consisted of 9 questions and were distributed to all of the EDs nationwide and received from June 2011 to July 2011. The data were analyzed using a Microsoft Excel spreadsheet (Microsoft, Redmond, WA, USA).

## Results

A total of 64 ED directors completed questionnaires among the 95 hospitals surveyed. The response rate was 65%. The contents of the questionnaires and results are shown in the tables. The characteristics of the hospitals from which directors responded are shown in Table 1. About half responded that trauma cases accounted for more than 15% of all ED visits. Most trauma patients were young males. Table 2 illustrates the current status of tetanus prophylaxis in Korean EDs. Two thirds of the EDs surveyed reported applying tetanus prophylaxis guidelines to wound management of more than 80% of trauma patients. However, about 45% of EPs considered giving tetanus booster vaccinations to less than half of the wounded patients and there were no differences in tetanus booster rates among different age groups. The reasons for not considering administration of tetanus-diphtheria vaccine to wounded patients are shown in Table 3. Most of the EPs were familiar with tetanus prophylaxis guidelines for wound management. However, more than half of the ED directors reported that the major reason for not considering tetanus-diphtheria vaccination was the assumption that patients already had tetanus immunity. Only 35.3% of ED directors reported that the major reason for not using tetanus-diphtheria vaccine is patient refusal. If a patient were to refuse the tetanus-diphtheria vaccination, the reason was the price of the vaccine rather than patient lack of understanding.

**Table 1:** Characteristics of Korean emergency departments

**Total number of responding EDs/ total number of EDs sent questionnaire (%)**	**Annual number of patient visits**
**64/95 (65)**	42242.2 ± 18633.7
Trauma patients as percentage of total ED patients	Frequency of response (%)
< 5%	0 (0)
5-10%	7 (10.9)
10-15%	27 (42.2)
> 15%	30 (46.9)
Male to female ratio for trauma patients	Frequency of response (%)
Male = Female	14 (21.9)
Male > Female	50 (78.1)
Male < Female	0 (0)
Most common age group of trauma patients (years)	Frequency of response (%)
10-25	4 (6.3)
26-40	47 (73.4)
41-54	8 (12.5)
>55	5 (7.8)

*ED* emergency department.

**Table 2:** Current status of tetanus prophylaxis in Korean emergency departments

**What percentage of clinicians in your department applies tetanus prophylaxis guidelines in wound management?**	**Frequency of response (%)**
<20%	3 (4.7)
20-50%	8 (12.5)
50-80%	12 (18.8)
>80%	41 (64.1)
What percentage of wounded patients receives tetanus-diphtheria vaccine?	Frequency of response (%)
10-20%	7 (10.9)
20-30%	8 (12.5)
30-50%	14 (21.9)
>50%	35 (54.7)
What is the most common age group that your clinicians consider for tetanus-diphtheria vaccination? (years)	Frequency of response (%)
10-25	2 (3.1)
26–40	23 (35.9)
41–54	24 (37.5)
>55	15 (23.4)

**Table 3:** Reasons for not considering tetanus-diphtheria vaccination in wounded patients

**Why don’t your clinicians consider administering tetanus-diphtheria vaccine to wounded patients?**	**Frequency of response (%)**
They do not know the tetanus prophylaxis guidelines	0
They assume that the patient has tetanus immunity	20 (58.8)
Refusal by the patient	12 (35.3)
The ED does not provide tetanus vaccination	2 (5.9)
Why do you think patients refuse tetanus- diphtheria vaccination?	Frequency of response (%)
Lack of understanding about tetanus	18 (34.0)
Price	35 (66.0)

*ED* emergency department.

## Discussion

Due to recent widespread immunization programs, the majority of tetanus patients in developed countries are elderly. Since the 1990s, the annual incidence of tetanus in the United States has been about 40 cases per year. Most cases of tetanus occur among adults with inadequate or unknown tetanus vaccination histories [10-12]. In Sweden, about 100 cases of tetanus were reported per year until the 1950s. The incidence has declined since the 1960s and the average annual incidence in the 1990s was fewer than 10 cases [13]. Germany’s history of tetanus prevalence is similar to Sweden’s [14].

As people age, tetanus immunity decreases. Stark et al. [14] reported that only 56% of people over 50 years of age in Germany have tetanus immunity. About 50% of Americans over 60 years of age have tetanus immunity, while only 27.8% of those over 70 years of age have immunity [12,15]. 80% of Swedish people less than 50 years of age had tetanus immunity, but again, only 27.8% of those older than 70 years of age had immunity [16].

In South Korea, the number of tetanus and infantile tetanus patients has also decreased remarkably, as the DTaP vaccination coverage rate in children has been over 90% since 1988 [3]. The Korea Centers for Disease Control and Prevention reported that the annual incidence of tetanus nearly disappeared in South Korea from 1980 to 2000; however, there have been about 10 tetanus cases per year since 2000 [9]. Shin et al. reported a total of 17 cases in a single 900-bed hospital over the course of 21 months. Although most of the patients were advanced in age, quite a few young adults, including a pregnant woman in her 20s, were also afflicted [17]. This epidemiologic feature is different from that of other developed countries. The tetanus immunity of Koreans is decreased in patients over 20 years of age, and only 10% of Koreans over 40 years old have tetanus antibody levels over 0.1 IU/mL [8]. This situation may be due to the late launch and relatively limited use of the tetanus-diphtheria vaccine in South Korea.

Tetanus prophylaxis in wound management is a major issue for EPs. According to tetanus prophylaxis wound management protocols, even patients with clean minor wounds should receive tetanus-diphtheria vaccinations if their last boosters were over 10 years prior [7]. Although Korean EPs are well informed regarding tetanus prophylaxis guidelines in wounded patients, tetanus-diphtheria booster vaccination rates have remained low and the EPs did not seem to consider differences in tetanus immunity according to age. This may be due to clinicians’ lack of awareness regarding the epidemiology of tetanus immunity.

The tetanus-diphtheria vaccine is safe and has fewer side effects than the DTaP vaccine when given as a booster. Possible side effects include fever, fatigue, headache, and lymphadenopathy. However, these side effects subside spontaneously within 2 or 3 days [18]. Factors that contribute to adverse reactions are age of the subject, route and method of injection, tetanus antitoxin levels prior to vaccination, and the presence of adjuvant [19-23]. The side effects of tetanus-diphtheria vaccination were previously studied in Koreans, and no major adverse reactions were reported [24].

Nevertheless, it may cause side effects and redundant costs to give patients with sufficient immunity a tetanus vaccination. EPs can obtain a tetanus vaccination history from patients themselves. However, this could be unreliable in some cases because patients do not always accurately recall their vaccination history. In addition, there is no system for hospitals and patients to assess their personal immunization records in South Korea. To solve such problems, Hatamabadi et al. has suggested that the use of point-of-care testing (POCT) such as the Tétanos Quick Stick (TQS; Nephrotek Laboratory, Rungis, France) could assess vaccination status more accurately than a structured medical interview [25].

Concerning the current status of tetanus prophylaxis in Korean EDs and the epidemiology of tetanus immunity, attitude changes on the part of clinicians should be urged and the promotion of tetanus immunity should be encouraged. Associated societies should provide ongoing member awareness campaigns on tetanus epidemiology for clinicians.

The South Korean government partially covers the cost of vaccinations in infants and children. However, adult patients have to cover the entire cost of tetanus-diphtheria vaccinations. This study showed that the reason for refusal of tetanus-diphtheria vaccination is price rather than lack of understanding. Thus, it can be concluded that if the government were to cover the cost of tetanus-diphtheria booster vaccinations, the vaccination rate would increase. Also, if patients were more informed about tetanus immunity, resistance due to price might decrease. These efforts may lead clinicians to use the tetanus booster vaccine more widely.

As clinicians are frequently confronted with patients that are at high risk for tetanus infection in emergency situations, they must be more thoroughly informed regarding the epidemiology of tetanus immunity and encouraged to use the tetanus-diphtheria booster vaccine after obtaining tetanus vaccination histories from patients or to use POCT.

There are some limitations to this study. One limitation is that all of the data are self-reported and thus could have been affected by recall bias. The data of this study are mostly based on broad estimates of the number/proportion of patient/ED characteristics. Another limitation is that we did not consider nonresponse bias, which exists in all questionnaire studies.

## Conclusion

Concerning the current status of tetanus prophylaxis in Korean EDs and the epidemiology of tetanus immunity, attitude changes should be encouraged among EPs regarding tetanus prophylaxis. As EPs are frequently confronted with patients that are at a high risk for tetanus infection in emergency situations, they need to be more informed regarding tetanus immunity epidemiology and encouraged to administer tetanus booster vaccines.

## Abbreviations

CDC: Centers for diseases control and prevention; EP: Emergency physician; ED: Emergency department; DTaP: Diphtheria and tetanus toxoids and acellular pertussis vaccine; Tdap: Tetanus toxoid with lower doses of diphtheria and acellular pertussis than DTaP.

## Competing interests

The authors declare that they have no competing interests.

## Authors’ contributions

YHY performed data analysis and drafted the manuscript. SWM developed the questionnaires and made critical revisions to the manuscript. JYK and YDC managed the data and critical revisions to the manuscript. YHK and SHC conceived the research and drafted the manuscript. Each author has read and approved the final manuscript.
